# Comparison of graph and animation: An unbalanced battle over two decades

**DOI:** 10.3389/fpsyg.2022.810557

**Published:** 2022-09-23

**Authors:** Qianhong Zhuang, Xiaobin Liu

**Affiliations:** School of Foreign Studies, South China Normal University, Guangzhou, Guangdong, China

**Keywords:** animation, picture, visualization, instructional, factor, cognitive

## Abstract

Numerous studies have produced contradictory findings about whether static or animated format is the better instructional tool. With a comparison between graphs and animations that has a genuine impact on learning and teaching, this review provides a comprehensive examination of (a) the theoretical foundations of visualized learning, (b) influencing factors, and (c) prospective future studies.

## Introduction

Scholars and practitioners alike frequently use the term “picture,” sometimes known as “graph,” “image,” and “diagram.” Animation is “an application that generates a series of frames so that each frame appears as an alteration of the previous one, and where the sequence of frames is determined, either by the designer or the user” (Bétrancourt and Tversky, 2000). A graph is occasionally referred to as a static graph, and an animation as a dynamic system when contrasting the two types.

The attempt to include graphs or animations into learning and teaching has been the subject of a protracted dispute. Previous investigations (such as Koroghlanian and Klein, 2004; Castro-Alonso et al., 2014; Ganier and de Vries, 2016; Andrä et al., 2020) came to conflicting conclusions. A few systematic reviews of these studies have been conducted, despite the fact that there are many studies that address various elements of their efficacy (see the review by Tversky et al., 2002, and the meta-analysis by Höffler and Leutner, 2007). Researchers have recently focused on identifying the mechanism underlying the disparate learning outcomes of these two visualization forms. The current mini-review aims to complement the previous studies and provide an in-depth analysis of (a) the theoretical underpinnings of visualized learning, (b) influencing factors, and (c) potential future directions.

## Cognitive theory of multimedia learning

The Cognitive Theory of Multimedia Learning is the fundamental theory that underpins visualized learning. Within the framework, Dual-channel Principle (Paivio, 1986) has stated that the human learning system separates auditory and visual input into distinct channels. Working memory’s processing and storage constraints have long been known to prevent it from handling numerous new elements at once or for a considerable amount of time (e.g., Miller, 1956; Peterson and Peterson, 1959; Cowan, 2001). Distributing the processing load to dual sensory channels will boost working memory capacity in comparison to just visual processing (Paivio, 1986; Moreno and Mayer, 1999). Therefore, it is very likely that using audio to explain graphics will result in greater transfer learning.

To address a rookie learner’s lack of knowledge in a particular subject where learners can digest information through dual channels, a graph or animation is frequently equipped with spoken expression or textual narration in practical teaching and learning. People learn more effectively from words and visuals than from just words, for instance. Contrary to reconstructing the complete process from a collection of still photos, one would think that animation helps conceptualize the process and so minimizes cognitive demands.

However, practitioners should be aware of two rules when employing animation in addition to text or voice.

First, it is conceivable that it is important to consider whether the visualization represents the material to be taught, that is, whether it serves as a presentation tool or is purely ornamental. The cognitive load theory states that learning through animations can lessen cognitive demands because the entire process is immediately perceptible. However, decorative visualization that offers extra information unrelated to learning objectives will put learners under needless cognitive load and strain their working memory (Kalyuga and Sweller, 2014) and this effect varies based on the students’ aptitude or prior knowledge. For example, the animation of “turning two commas into two handles and removing the non-restrictive attributive clause between the two commas” can, for instance, help students better understand the role of the non-restrictive attributive clause as supplementary explanation when teaching “non-restrictive relative clause.” On the other hand, some ornamental animations, like a moving flower on the instructor’s slides, will add extra cognitive stress. As a result, the audio content should be carefully picked to closely match any modifications or visual components (Moreno and Mayer, 2000). Otherwise, the visual and aural components overload the working memory.

Secondly, having animation requires a higher level of working memory due to the ephemeral nature of animation. Prior to further processing, the rich and fleeting information taken from the animation must be digested and kept by the working memory, or there is a risk of missing crucial information and task failure (Ainsworth and VanLabeke, 2004). For instance, newcomers to the field may find it challenging to comprehend the animation’s quick changes (Kalyuga et al., 2003). Some instructors will play films or animations during the warm-up period of a brand-new class. It is difficult without prior knowledge if students want to learn something through animation. Thus, if the educator’s goal is to let the learner pay attention to the details in the animation, it’s suggested that play the animation several times or slow down the animation speed, let students pay attention to certain issues intentionally before watching animation, use prominent colors or arrows in the animation to emphasize key points, and use pictures together to give students time to observe and think carefully.

## Embodied cognition theory

Embodied Cognition Hypothesis is a different theory that has a strong connection to visual learning. The core tenet of embodied cognition is that we perceive things in the environment not as abstract symbols but rather as physical realities (Barsalou, 1999). The creation of a kinematic analogue mental model is a result of cognitive operations that make use of sensorimotor resources (Wilson, 2002; Borghi and Pecher, 2011; Johnson-Glenberg et al., 2016). For instance, because evolution has enabled modern humans to master these essential movements, they are adept at learning high embodiment tasks, particularly those involving body and hand movements (Castro-Alonso et al., 2016). Notably, the purpose of the visual presentation is to act as a stimulus and aid in creating an internal representation of the external environment that the students are mentally emulating.

In this sense, domain or topic can affect visualized learning outcomes. Physics (H’mida et al., 2020) and biology (Koć-Januchta et al., 2020) are domains that researchers have discussed the most widely by attaching importance to the animation advantage in understanding complex systems. Since it displays changes over time, the animation is assumed to be conducive to understanding and remembering dynamic systems (e.g., STEM), such as biological processes, natural phenomena, or mechanical equipment (Berney and Bétrancourt, 2016). In line with this, it should come as no surprise that spatial visualization skills—the capacity of students to picture modifications in an object’s structure as it rotates in two or three dimensions—affect each student’s learning outcomes. Depending on students’ spatial abilities, employing animation has different advantages (Yang et al., 2003). Less talented pupils frequently struggle to comprehend how visual–spatial representations work (Hegarty, 2014). Compared with mathematics, physics, and mechanics, the beneficial effects of animation are not found systematically in meteorology, biology, and history (Schneider, 2007).

Additionally, animations are recommended since they are more efficient than photos when it comes to executing manual tasks and teaching and acquiring procedural knowledge (Höffler and Leutner, 2007; Ayres et al., 2009). Similar to this, it was demonstrated that animations have a greater influence on procedural knowledge than conceptual or declarative knowledge (Höffler and Leutner, 2007).

It is strongly advised that learners mimic the movements while watching the animation when teaching a procedural concept that they can replicate. Due to the activation of specialized visual and motor areas, Mayer (2005)‘s neuroimaging findings showed that learning is more effective when gestures are made. Similarly, Demir-Lira et al. (2020) discovered that hand gestures used in conjunction with speech outperformed visual cues displayed on a screen.

It makes the case that metacognitive and emotional elements may combine to have an impact when imparting non-procedural knowledge. While emotional elements mediate learning by boosting the learner’s cognitive engagement, which results in gains, metacognitive factors mediate learning through controlling cognitive and affective processes (McGuinness, 1990; Morris, 1990; Moreno et al., 2001; Park et al., 2011; Kim et al., 2020). It is advised to involve learners’ emotion and cognition when using images to communicate non-procedural knowledge. Instead of utilizing animations and images to depict the “shy look,” as an example, when learning the word “shy,” we advise using them to demonstrate the process of “the little girl is very shy when she sees her mother’s colleague on the road and wants to say hello.”

What’s more, practitioners are encouraged to incorporate visually appealing elements like colors, shapes, texture, shades, lights and other visual characteristics to pique learners’ interest and motivation (e.g., Sanchez and Wiley, 2006; Mayer and Estrella, 2014). However, the Cognitive Theory must guide all endeavors. For less gifted students, seductive elements can occasionally result in cognitive overload (Moreno and Park, 2010; Magner et al., 2013).

## Discussion

It is important to inform upcoming researchers and practitioners about the existing taxonomy and instances of these confounding variables. Line drawings and video-based animation were contrasted in Christian Andrä et al.’ (2020) study, which produced an imbalanced level of realism. The study by Akinlofa et al. (2014) was biased in favor of animation because only animation offers buttons to adjust pace. Only the animated version of the photo-realistic words was used in Luzón and Letón (2015). Hand-drawn items were visible in the whiteboard animation, but the hands were absent from the still image (Türkay, 2016).

Dynamic enrichment presents information that is not available in the static versions, which is the current and serious issue in the field. Some conclusions become doubtful due to the lack of comparability. Over the past 20 years, those conclusions have misidentified the static or animated format as the more advised instructional method in this subject. In conclusion, animation did not significantly outperform static graphs when some moderators were in place. Avoid contrasting apples and oranges; retain the information balance that the two visualization formats give, according to Castro-Alonso et al. (2016). Previous studies’ inconsistent findings warrant more meticulously planned research that takes into account all the moderating variables at play.

The main distinction between an animation and an image is whether or not they change. Changes in space and time can be plainly conveyed using animation. Animation, according to Ploetzner and Lowe (2012), should be composed of modelled entities and should visualize changes over time. However, for a very long time, the idea was confusedly applied with slide shows, films, and simulations with graph interfaces. In this situation, it is crucial to reevaluate a clearer definition of animation. We advise separating animation from other dynamic representations by pointing out two characteristics: (a) it exhibits perceptible change; and (b) it uses modelled entities to display information rather than still images or moving pictures.

Researchers are urged to place a high value on applying this delicate animation theory to teach dynamic processes like learning movements. Implicitly, there is still a ton of research room in the areas of verb learning, evolution, and the creation of animated stories.

## Author contributions

QZ and XL have approved it for submission to Frontiers in Psychology. All authors contributed to the article and approved the submitted version.

## Conflict of interest

The authors declare that the research was conducted in the absence of any commercial or financial relationships that could be construed as a potential conflict of interest.

## Publisher’s note

All claims expressed in this article are solely those of the authors and do not necessarily represent those of their affiliated organizations, or those of the publisher, the editors and the reviewers. Any product that may be evaluated in this article, or claim that may be made by its manufacturer, is not guaranteed or endorsed by the publisher.
